# Pulmonary Tuberculosis Following Immune Checkpoint Inhibitor Treatment for Recurrent Maxillary Squamous Cell Carcinoma

**DOI:** 10.7759/cureus.53203

**Published:** 2024-01-29

**Authors:** Shogo Kikuta, Yushi Abe, Katsumi Shinozaki, Naoko Seki, Jingo Kusukawa

**Affiliations:** 1 Dental and Oral Medical Center, Kurume University School of Medicine, Kurume, JPN

**Keywords:** pembrolizumab, oral cancer, immune checkpoint inhibitors, immune-related adverse events, mycobacterium tuberculosis

## Abstract

Immune checkpoint inhibitors (ICIs) like nivolumab and pembrolizumab are effective treatments for recurrent/metastatic squamous cell carcinoma of the head and neck (R/M SCCHN). However, they can lead to immune-related adverse events (irAEs) and tuberculosis (TB) reactivation. We present a case of a 79-year-old male with recurrent maxillary squamous cell carcinoma treated with pembrolizumab, cisplatin, and 5-fluorouracil. The patient developed a fever, and pulmonary TB development was confirmed. Prolonged TB treatment was required, and ICI treatment was discontinued. The patient ultimately opted for palliative care due to aggressive tumor growth. TB development during ICI treatment is a rare but important concern, especially in TB-endemic areas. Vigilant monitoring and screening might be essential to manage this risk in cancer patients with R/M SCCHN receiving ICIs.

## Introduction

Immune checkpoint inhibitors (ICIs), encompassing anti-programmed death-1 (PD-1) as well as PD-L1 and PD-L2 agents, along with cytotoxic T-lymphocyte-associated antigen 4 (CTLA-4) signaling blockade, have demonstrated efficacy across a spectrum of malignancies [1,2]. Grounded in the outcomes of two pivotal clinical trials, namely the CheckMate 141 study and the KEYNOTE-048 study, two anti-PD-1 pharmaceuticals, nivolumab and pembrolizumab, have emerged as therapeutic options for recurrent/metastatic squamous cell carcinoma of the head and neck (R/M SCCHN) [3,4]. It is noteworthy that ICIs possess the potential to incite immune-related adverse events (irAEs), a profile distinct from traditional chemotherapeutic agents. In recent years, there has been a burgeoning corpus of literature documenting the reactivation of tuberculosis (TB) during the course of ICI administration [2,5]. Herein, we present a singular case wherein the development of pulmonary TB ensued subsequent to the initiation of ICI for the management of recurrent maxillary squamous cell carcinoma.

## Case presentation

**Figure 1:**
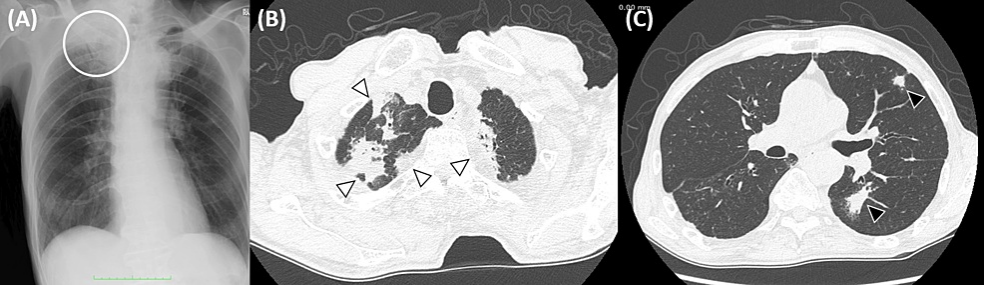
Chest radiographic and high-resolution CT images A) Chest radiography revealed an abnormal opacity of the right upper lobe lung (white circle). B, C) Axial high-resolution CT images of the chest revealed consolidations with cavitation in both upper lobes (white arrowheads) and solitary pulmonary nodules in the anterior segment and the left superior segment of the left lung (black arrowheads).

A 79-year-old man had previously undergone three partial maxillectomies, modified radical neck dissection, and concurrent chemoradiotherapy with a cumulative cisplatin dose of more than 200 mg/m^2^ for maxillary squamous cell carcinoma on the left side in our department. Subsequently, the patient revisited our department for consideration of partial maxillectomy for recurrent maxillary squamous cell carcinoma. The patient had no past medical history of tuberculosis and no smoking, immunosuppression situation, diabetes, or obesity. Histopathological analysis of the excised tissue unveiled a positive surgical margin. Immunohistochemical staining of the excised tissue was employed to probe the PD-L1 expression, which yielded a combined positive score (CPS) of 20. For the management of recurrent and recalcitrant squamous cell carcinoma of the craniofacial region, a regimen consisting of pembrolizumab (200 mg/body), cisplatin (80 mg/m²), and 5-fluorouracil (800 mg/m² per day for four consecutive days) was intravenously administered at three-week intervals. The patient underwent no TB screening prior to the drug administration. Following two cycles of this immuno-chemotherapeutic regimen, the patient developed pyrexia exceeding 38 degrees Celsius despite obtaining negative results on COVID-19 reverse transcription polymerase chain reaction (RT-PCR) and rapid influenza tests. Subsequent chest radiography and computed tomography (CT) scans disclosed nodular radiographic manifestations suggestive of pneumonia within the upper right and lower left pulmonary fields (Figure 1).

**Figure 2:**
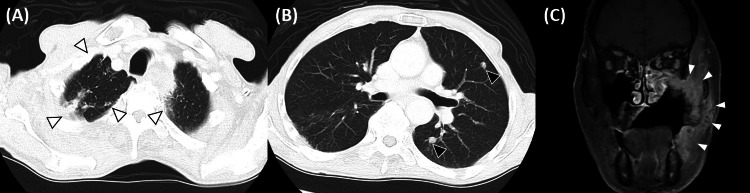
High-resolution CT and MRI images A, B) High-resolution CT of the chest after eight months of anti-tuberculosis (TB) treatment represented the decrease of opacities and old inflammatory change after TB (white and black arrowheads). C) Contrast-enhanced magnetic resonance imaging (MRI) of the head and neck region revealed the presence of an aggressive tumoral formation, extending from the left maxillary region to the masticatory muscle space, pterygopalatine fossa, suborbital fossa, accompanied by localized recurrence (white arrowheads).

Consequently, a bronchoscopy was performed at the Department of Respiratory Medicine in our institution. Specimens procured from the apical region of the right lung exhibited positive outcomes in dry smear microscopy (Gaffky 1). Sputum samples underwent mycobacterial smear microscopy, culture, and nucleic acid amplification. All diagnostic tests yielded affirmative results, ultimately culminating in the diagnosis of pulmonary TB. The patient was immediately transferred to an isolation ward in another institute for TB treatment and received anti-TB medication, including rifampicin, isoniazid, pyrazinamide, and ethambutol. Following a three-month hiatus prompted by a cutaneous eruption induced by the TB drug, isoniazid monotherapy was reinstated in conjunction with levofloxacin and streptomycin. Throughout TB treatment, the patient experienced a dearth of comprehensive examinations for oral cancer after ICI therapy, attributable to the absence of an oral and maxillofacial surgeon at the transferred hospital. Seven months after his transfer, the patient's quarantine status was rescinded. The patient subsequently presented at our department due to the emergence of aggressive neoplastic growth, accompanied by necrotic tissue, spanning from the median aspect of the palate to the left buccal mucosa. Radiographic assessment via chest CT indicated a reduction in the previously observed pulmonary infiltrates, transforming into nodular opacities with comparably homogeneous perimeters, suggesting old inflammatory changes after pulmonary TB (Figure 2A, B).

Contrast-enhanced magnetic resonance imaging (MRI) of the head and neck region unveiled the presence of an aggressive tumoral formation, extending from the left maxillary region to the masticatory muscle space, pterygopalatine fossa, suborbital fossa, accompanied by localized recurrence (Figure 2C). The patient and his family expressed a reluctance to pursue any further therapeutic interventions beyond this point. Consequently, a collective decision was reached to furnish the patient with the utmost in palliative care. One year after the diagnosis of TB, the patient died due to a nutritional disorder following tumor progression.

## Discussion

Mycobacterium tuberculosis (TB) imposes a formidable global health burden, with approximately 1.7 billion individuals harboring latent TB infections worldwide [6]. TB constitutes a significant malady of global import, with an annual incidence of nine million new cases and a mortality rate of 1.5 million per annum [2,7]. Supplementary risk factors for TB infections encompass smoking, immunosuppression, diabetes, obesity, residency or travel in areas, inhabiting overcrowded environs, suboptimal nutritional status, and male gender [7]. Indeed, TB has earned the appellation of a disease transcending geographical boundaries, and notwithstanding its historical association with developing countries, mass human migration has effectively dispelled any notion of its confinement to these areas [8,9].

Cancer represents an intricate and multifarious consortium of maladies distinguished by the unbridled proliferation and division of aberrant cellular entities within the corporeal domain. It assumes paramount significance as a profound global health preoccupation, emerging as a preeminent etiology of mortality on a planetary scale. Immunotherapy, exemplified by ICIs, embodies a paradigm of oncological intervention harnessing the innate components of the immune system to combat neoplastic entities [10]. Immunotherapeutic modalities, whether employed in isolation or as adjunctive measures alongside conventional therapeutic regimens encompassing radiotherapy and chemotherapy, have attained substantial accolades as quintessential therapeutic modalities for a multitude of malignancies, including oral cancer [11]. On the other hand, by potentiating the immunological response, immune checkpoint blockade can engender inflammatory manifestations, denoted as irAEs. While irAEs have the capacity to affect virtually any organ system, their predilection typically encompasses the gastrointestinal tract, endocrine glands, integumentary system, and hepatic milieu [12]. Infrequently, the central nervous system, as well as the cardiovascular, pulmonary, musculoskeletal, and hematologic systems, may become implicated. Given the extensive spectrum of potential irAEs, their proficient management necessitates a collaborative, multidisciplinary approach involving healthcare providers spanning the entirety of the clinical continuum [13]. An expanding body of literature has recently surfaced, documenting instances of tuberculosis reactivation occurring concomitantly with ICI administration [2,5]. To the best of our knowledge, there have been some reports of TB reactivation following ICI therapy, including ipilimumab, nivolumab, pembrolizumab, and atezolizumab (Table 1) [5,14-29].

**Table 1:** Summary of case reports with tuberculosis reactivation after administration of immune checkpoint inhibitors TB - tuberculosis; ICI - immune checkpoint inhibitor; NSCLC - non-small cell lung cancer; SCC - squamous cell carcinoma; RFP - rifampicin; INH - isoniazid; EB - ethambutol; PZA -pyrazinamide; SM - streptomycin;

Author	Year	Age	Sex	Ethnicity	Cancer	ICIs	Number of administrations	Outcome of ICIs	ICI reinitiation	Previous therapy	TB treatment	Patient survival status
Lee et al. [14]	2016	87	Male	Asian	Hodgkin's lymphoma	Pembrolizumab	5	Discontinuation	Not specified	ABVD chemotherapy, radiotherapy, clorambucil+prednisolone, gemcitabine+oxaliplatin, brentuximab vedotin	REF, INH, EB, PZA	Not specified
Fujita et al. [15]	2016	72	Female	Asian	Advanced NSCLC	Nivolumab	8	Discontinuation	Not specified	Carboplatin+docetaxel, carboplatin+gemcitabine	Not specified	Not specified
Chu et al. [16]	2017	59	Male	Asian	Metastatic NSCLC	Nivolumab	3	Skipped one cycle	Yes	Gefitinib, other unspecified chemotherapy	Not specified	Not specified
Jensen et al. [17]	2018	56	Male	Caucasian	Metastatic NSCLC	Nivolumab	12	Discontinuation	Not specified	Unspecified chemoradiotherapy, pemetrexed	Not specified	Not specified
Picchi et al. [18]	2018	50	Male	Caucasian	Metastatic Melanoma	Pembrolizumab	4	Continuation	-	None specified	4-drug regimen	Alive
		64	Male	Caucasian	Metastatic NSCLC	Nivolumab	2	Discontinuation	No	None specified	4-drug regimen	Deceased
Tetikkurt et al. [19]	2018	53	Male	Not specified	SCC of oral cavity	Pembrolizumab	6	Discontinuation	Yes	Excisional biopsy, surgical resection, Cisplatin+radiation	Not specified	Alive
He et al. [20]	2018	65	Female	Asian	Advanced Melanoma	Pembrolizumab	10	Discontinuation	Yes	Surgical resection, high-dose IL-2	RFP, INH, EB, PZA, SM, moxifloxacin	Alive
Elkington et al. [21]	2018	62	Female	Not specified	Ocular Melanoma	Ipilimumab, Pmabrolizumab	Not specified	Not specified	Not specified	Surgical resection	Not specified	Not specified
Takata et al. [22]	2019	75	Male	Asian	Metastatic NSCLC	Nivolumab	15	Discontinuation	Yes	Carboplatin+pemetrexed, Carboplatin+albumin-bounded, paclitaxel, S-1+gemcitabine, palliative radiation	REF, INH, EB, PZA	Alive
Barber et al. [23]	2019	59	Male	Asian	Metastatic Nasopharyngeal cancer	Nivolumab	3	Not specified	Not specified	Not specified	RFP, INH, EB, PZA, SM	Deceased
		83	Male	Caucasian	Metastatic MCC	Pembrolizumab	12	Not specified	Not specified	Not specified	REF, INH, EB, PZA	Alive
Tsai et al. [24]	2019	49	Male	Not specified	SCC of hard palate	Nivolumab	6	Discontinuation	No	Cisplatin+radiation, cetuximab, paclitaxel, carboplatin	Not specified	Deceased
van Eeden et al. [25]	2019	56	Female	Caucasian	Metastatic NSCLC	Nivolumab	Not specified	Discontinuation	No	Gemcitabine+carboplatin, pemetrexed+radiation to hilar mass	REF, INH, EB, PZA	Deceased
Inthasot et al. [26]	2019	69	Male	Not specified	Metastatic lung adenocarcinoma	Nivolumab	18	Not specified	Not specified	Cisplatin+pemetrexed, maintenance pemetrexed	Not specified	Not specified
Anastasopoulou et al. [27]	2019	76	Female	Caucasian	Advanced melanoma	Nivolumab, Ipilimumab	8	Discontinuation	No	Interferon	REF, INH, EB, PZA	Deceased
		85	Male	Caucasian	Metastatic melanoma	Atezolizumab	9	Continuation	-	None	REF, INH, PZA	Alive
Lau et al. [5]	2021	29	Female	Asian	Metastatic Nasopharyngeal Ca	Pembrolizumab	Not specified	Discontinuation	Yes	Gemcitabine+cisplatin, cisplatin + 5-fluorouracil, gemcitabine and carboplatin, capecitabine, metronomic cyclophosphamide, cisplatin + radiation	REF, INH, EB, PZA	Alive
Murakami et al. [28]	2021	73	Male	Asian	Metastatic NSCLC	Pembrolizumab	5	Discontinuation	Yes	None	REF, INH, EB, PZA	Alive
Suliman et al. [29]	2021	58	Female	Not specified	Metastatic NSCLC	Pembrolizumab	6	Discontinuation	No	None	REF, INH, EB, PZA	Alive
Our case	2022	79	Male	Asian	Recurrent maxillary SCC	Pembrolizumab	2	Discontinuation	No	Surgical resection, cisplatin+radiation	RFP, INH, EB, PZA, SM	Deceased

There have been only three cases of TB development as irAEs in oral cancer, including the present case, and this is extremely rare [19,24]. In Tabe 1, 14 out of 21 cases (66.7%) opted for the discontinuation of ICI treatment after TB development. Interestingly, six cases resumed ICI treatment, with five of these cases (83.3%) maintaining an alive status. In the present case, the absence of medical practitioners proficient in oral cancer treatment at the patient's transferred healthcare facility hindered cancer treatment during TB treatment, resulting in tumor growth. Hence, the prognosis of cancer in such cases may hinge upon the feasibility of continued ICI treatment concurrently with TB management.

The containment of TB in its latent state is achieved through vigilant surveillance of TB-targeted CD4+ and CD8+ T cells, which incidentally serve as the focal points of ICIs [30]. It is noteworthy that the pathophysiology underlying TB reactivation subsequent to ICI therapy is intricate and remains inadequately comprehended, encompassing both innate and adaptive immune responses [2]. Within the purview of the innate immune response, exposure to TB precipitates an upregulation of programmed cell death protein 1 (PD-1) and programmed death-ligand 1 (PD-L1) expression on natural killer (NK) cells, a component of the innate immune system [2]. This, in turn, results in the liberation of interferon-γ (IFNγ) and subsequent NK cell-mediated cytotoxicity. Consequent interaction between PD-1 and its ligands, PD-L1 and PD-L2, may exert inhibitory effects on further NK cell activation, thus attenuating the potential for tissue damage caused by ongoing inflammation. Turning to the acquired immune response, TB can strategically exploit this arm of the immune system to evade host defenses by impeding IFNγ release and enhancing PD-1 expression, consequently suppressing CD8+ cell cytotoxicity [2]. Intriguingly, in vitro experimentation has demonstrated that the blockade of PD-1/PD-L1 interactions through ICI intervention augments CD8+ cell cytotoxicity against IFNγ-activated macrophages, thereby inciting TB reactivation [31]. It is paramount to recognize that PD-1/PD-L1 inhibition by ICIs has been substantiated in vitro as a mechanism potentiating CD8+ cell-mediated cytotoxicity against IFNγ-activated macrophages, ultimately leading to TB reactivation [2,5].

While guidelines have been disseminated for the screening of TB before the initiation of biological agents, such as anti-tumor necrosis factor-α (TNFα), in the context of various chronic inflammatory ailments such as rheumatoid arthritis [5,32], it is noteworthy that a consensus regarding the routine screening for TB before the administration of ICIs in cancer patients remains conspicuously absent, especially in low endemic areas. Recent retrospective investigations have unveiled that TB infection has been observed in a mere 0.1 to 1.67 % of patients following PD-1/PD-L1 therapy [33-36]. It is worth emphasizing that in areas characterized by a higher prevalence of TB, the incidence of such infections would undoubtedly be significantly elevated. In the present case, the patient domiciled in a low-endemic area underwent no TB screening prior to ICI treatment. Since latent TB infection is associated with TB development as irAEs, the present case may also have had it.

## Conclusions

In the present case at hand, TB developed subsequent to ICI treatment, resulting in the necessity for treatment interruption and the unwelcome resurgence of the malignancy. Despite the limited number of reported cases, it is incumbent upon us to underscore the imperative for a comprehensive appreciation of the potential for TB reactivation subsequent to the employment of ICIs in the treatment of R/M HNSCC.
